# Ganglion Recurrence Rates After a Simple Puncture and a Review of the Literature

**DOI:** 10.7759/cureus.84039

**Published:** 2025-05-13

**Authors:** Nathan C Winek, Mark Ren, Grace Kirkwood, Taylor Paskey, Robert J Strauch

**Affiliations:** 1 Orthopedic Surgery, Columbia University College of Physicians and Surgeons, New York, USA

**Keywords:** dorsal carpal ganglion, ganglion cyst aspiration, hand mass, mucous cyst, painless mass of hand, percutaneous aspiration, recurrent ganglion cyst, retinacular cyst, volar carpal ganglion

## Abstract

Aim

Ganglia are the most common masses of the hand. While benign, they can be symptomatic. Nonsurgical treatment includes observation, manual rupture, puncture, or aspiration. There is a broad range of recurrence rates cited in the literature with aspiration and puncture. The purpose of this study is to determine recurrence rates and patient satisfaction after a simple puncture of the hand and wrist ganglia.

Materials and methods

We retrospectively identified a cohort of consecutive new patients arriving at the senior author’s (RS) office from 2020 to 2022 who underwent simple puncture of a ganglion. Patients were examined by the senior author and the diagnosis of a ganglion was confirmed upon simple puncture with expression of mucinous fluid. A chart review was performed to collect basic demographic data, location of ganglion, and duration of symptoms. Patients were then contacted via telephone and their satisfaction and the rate of recurrence was determined.

Results

There were 64 patients identified as having undergone puncture of their ganglia. There were 34 patients able to be reached via telephone and 56% of the ganglia recurred with an average time to recurrence of 43 weeks. Of the 34 patients that participated, 79% (27/34) reported that they would choose simple puncture again if they had another ganglion and 76% (26/34) reflected favorably on the puncture procedure. Of the 19 patients with recurrence, eight patients underwent an additional procedure: four underwent surgery and four underwent repeat puncture, with a 75% recurrence rate in the repeat puncture group.

Conclusions

Our series demonstrates a recurrence rate of 56% for hand and wrist ganglia after simple needle puncture with no complications and high patient satisfaction. The literature has conflicting evidence regarding the optimal treatment of hand and wrist ganglia, however, a simple needle puncture is a reasonable first step in treating ganglia and can immediately confirm the diagnosis of a ganglion.

## Introduction

Ganglia are the most common masses found in the hand and wrist [1]. They tend to occur in the 2nd to 4th decades of life and more often in women than men [2,3]. Dorsal carpal ganglia, volar carpal ganglia, flexor tendon sheath ganglia otherwise known as reticular cysts, and distal interphalangeal joint ganglia otherwise known as mucous cysts are the most common ganglia in the hand and wrist [3,4]. Despite arising from various locations and structures, they histologically appear indistinguishable, suggesting a common underlying pathogenesis [5-7]. The main cyst of a ganglion is relatively acellular with fibrous tissue and lacking an epithelial lining that would be characteristic of a true cyst. As such, the term “ganglion cyst” is a misnomer and would be more aptly described as a pseudocyst. The term “ganglion cyst” has nevertheless become ingrained into our literature and nomenclature [7]. Patients typically present less so for pain and more for cosmetic reasons or concern about a growth [2]. In the 1700s Laurence Heister wrote in his surgical text: "The inspissated matter of a ganglion may be happily dispersed by rubbing the tumor well every morning with saliva…a cure may be readily performed if the patient frequently lays his hand on a table and strikes on the tumor with his fist…or a wooden mallet armed with lead. If none of these means prove effectual, it will necessary to remove the tubercle by incision…I have myself several times happily removed them this way" [8].

Three hundred years later, treatment options essentially remain the same: observation, aspiration or rupture, and surgical excision. For some patients, observation is not an adequate treatment avenue while surgical excision may be considered as a last resort. Various non-operative measures have been attempted in treating ganglia including manual rupture, suturing technique, puncture, and aspiration with or without injection of steroids or caustics [1,9-12]. These methods all more or less work in the same fashion by decompressing the main cyst by means of puncture or rupture with egress of the mucinous fluid without removal of the main cyst wall or its stalk. The rates of success with these vary significantly in the literature. In their review, Nahra and Bucchieri cited cure rates anywhere from 15-89% with aspiration [2]. This range in the literature is broad and would benefit from further investigation to better counsel patients and recommend in-office versus surgical procedures.

We regularly offer patients simple needle punctures of their ganglion with a manual expression of the mucinous fluid as a diagnostic and therapeutic first-line measure. We aim to describe this technique, review the recurrence rates, and evaluate patient satisfaction.

## Materials and methods

Patient selection

After institutional review board (IRB) approval, we retrospectively identified a cohort of consecutive new patients arriving at the senior author’s office from January 1, 2020, to December 31, 2022, who underwent puncture of a ganglion in the hand or wrist. The Columbia University IRB approval number was AAAV0805(M00Y01). Patients were contacted via a telephone call. Patients who declined to participate or were unreachable after three separate phone calls on separate days were excluded.

Procedural technique

**Figure 1:**
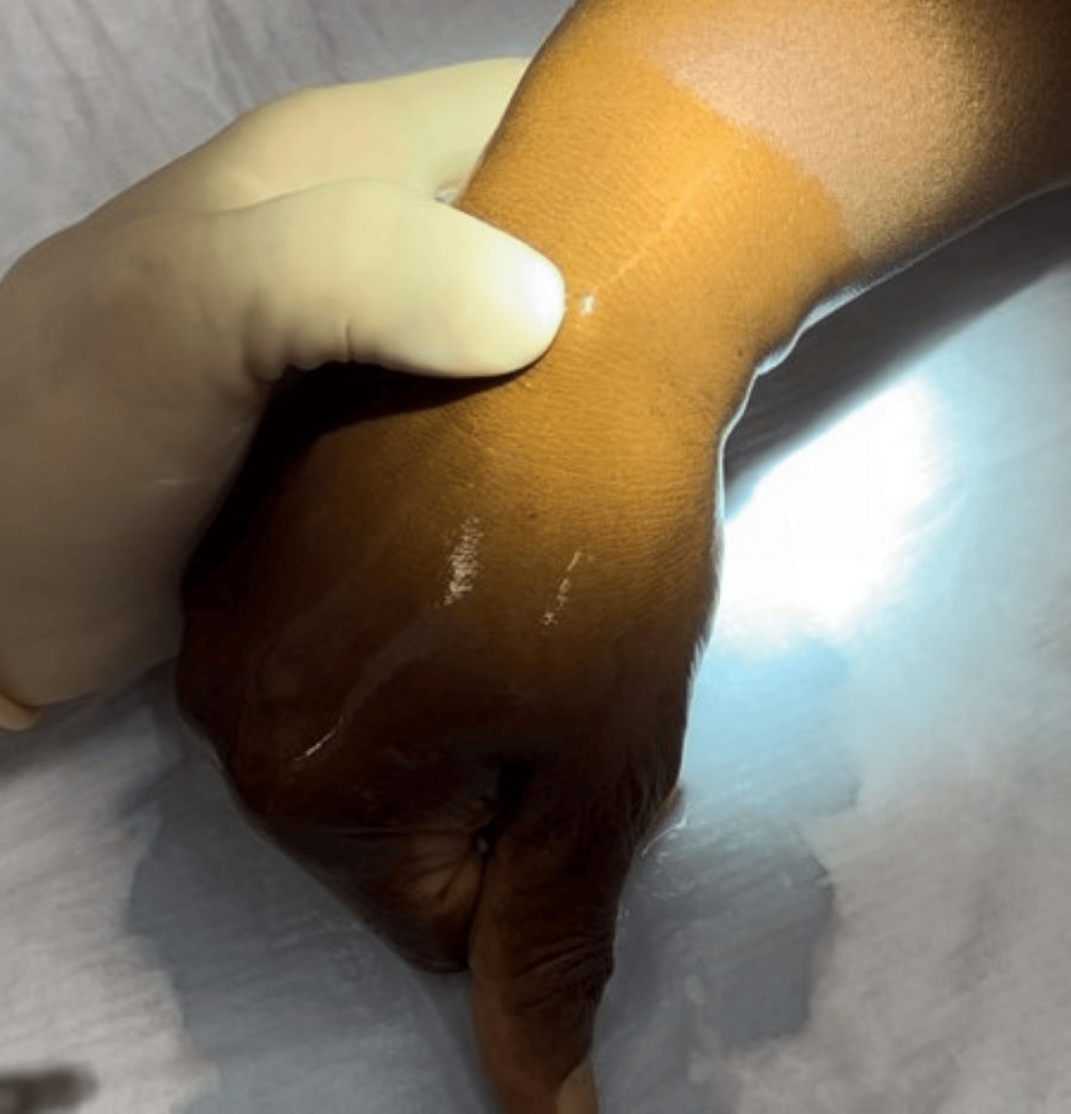
Dorsal carpal ganglion stabilized with nondominant hand

Patients were examined by the senior author and a working diagnosis of a ganglion was made based on the characteristic locations, appearance, and ability to transilluminate. The diagnosis was confirmed following needle puncture with expression of mucinous fluid. The physician’s nondominant hand is used to stabilize and firmly compress the ganglion (Figure 1) and then the needle is used to quickly puncture the ganglion.

**Figure 2:**
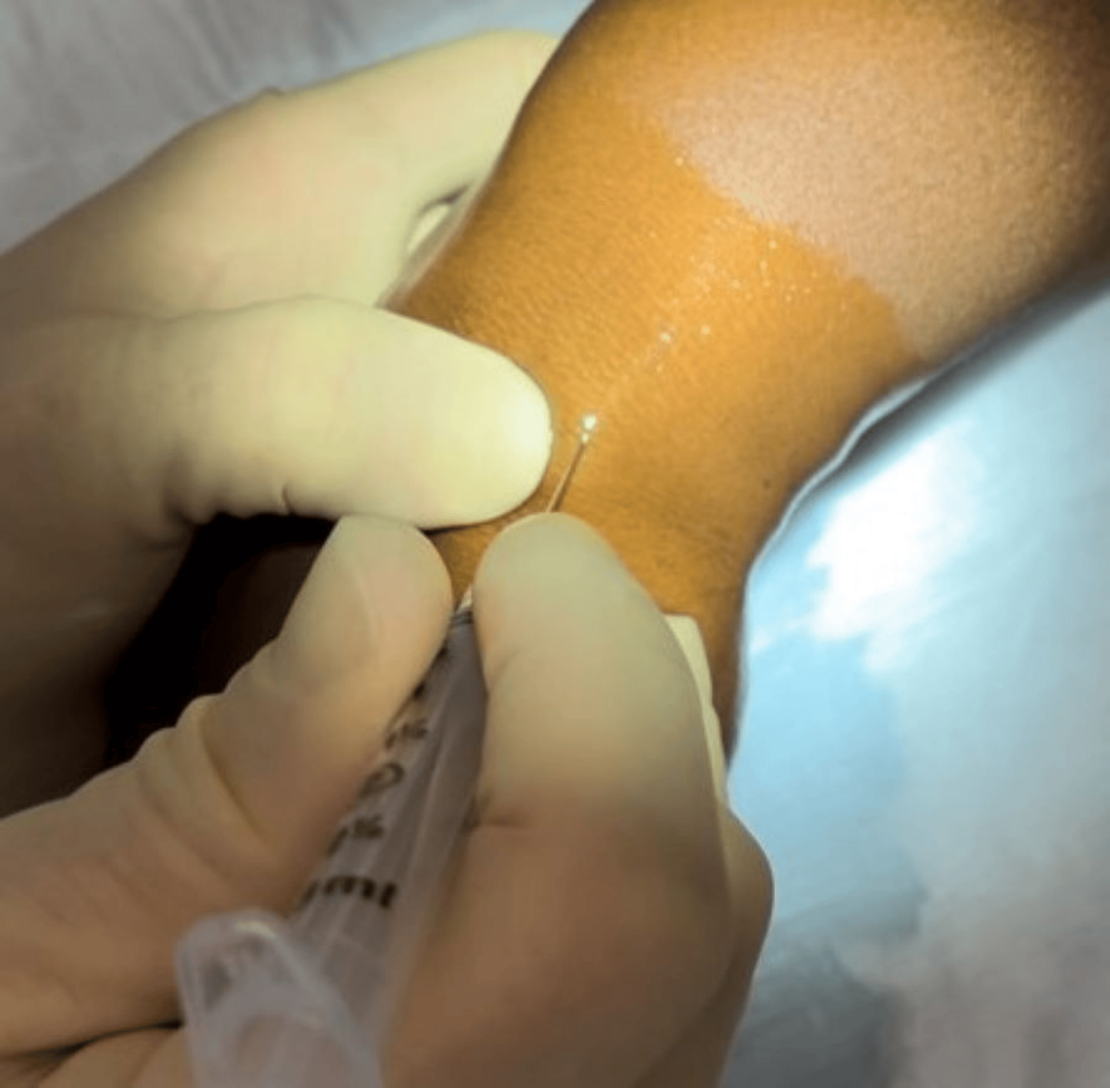
22-gauge needle inserted into the ganglion

The procedure is performed utilizing a 22-gauge needle on an empty 3 cc syringe for greater control of the needle (Figure 2). Alcohol is used to sterilize the skin.

**Figure 3:**
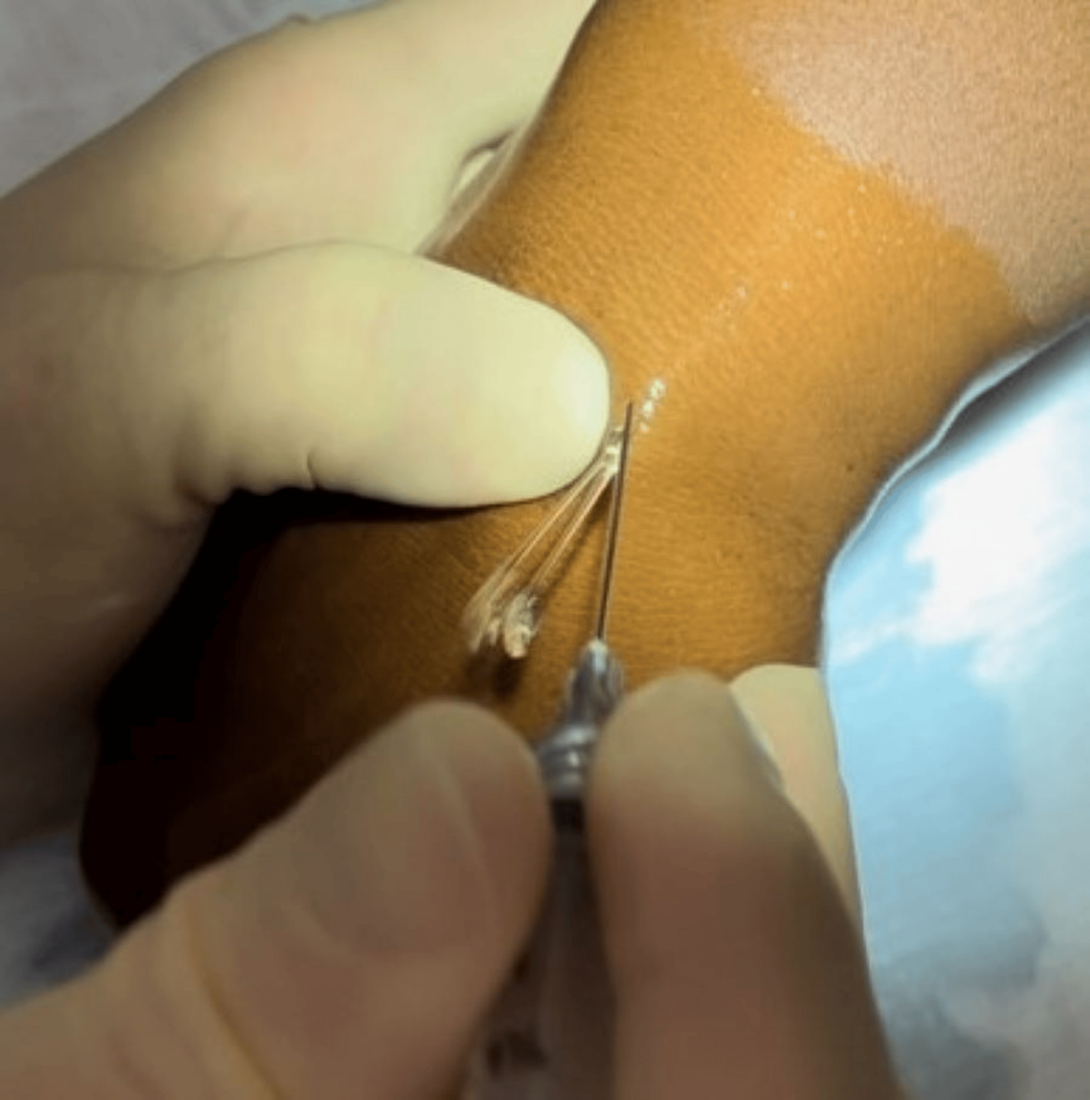
Expression of mucinous material with continued pressure from nondominant hand

**Figure 4:**
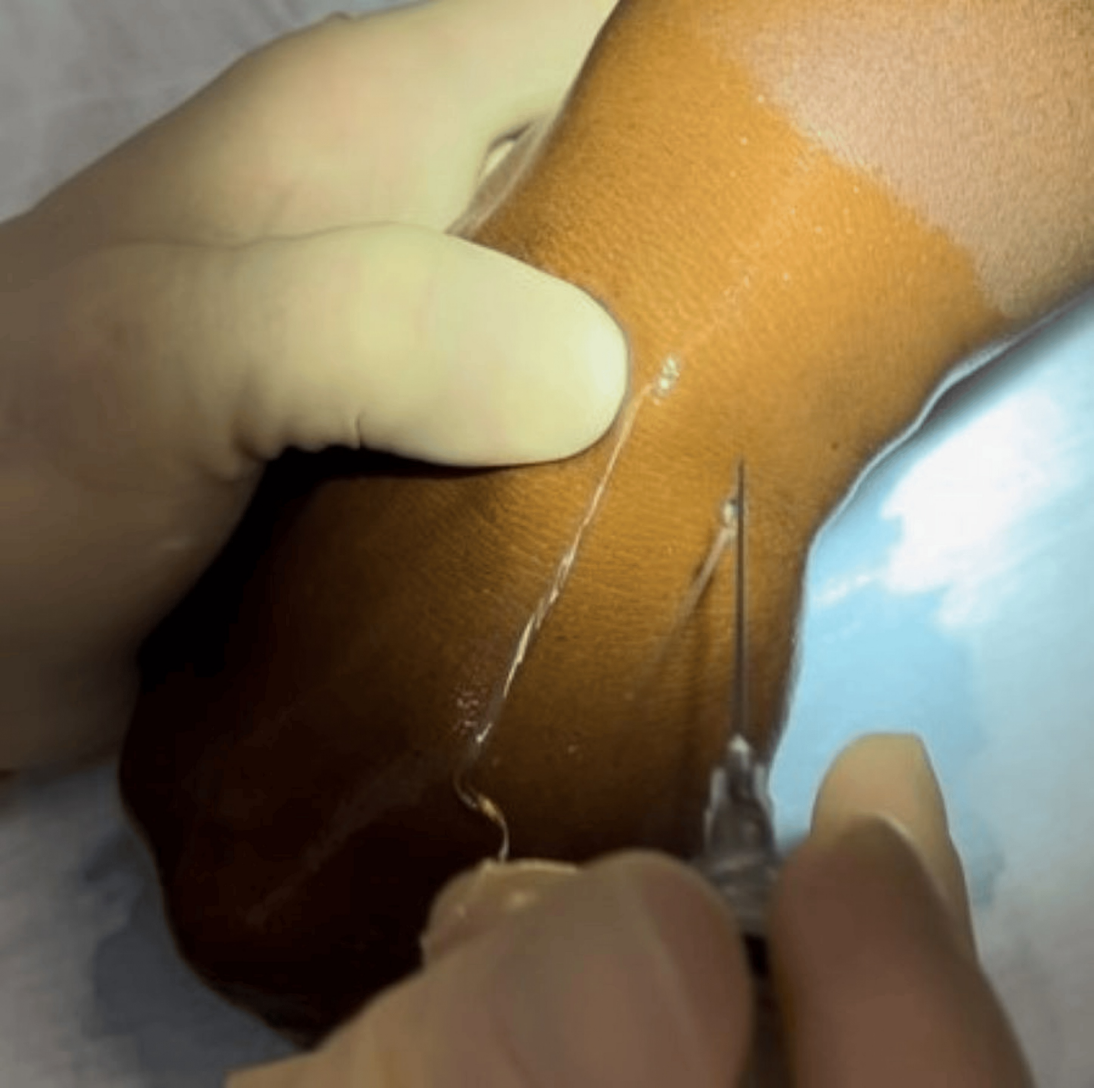
Further expression of mucinous material

The fluid from the ganglion cyst usually erupts either through the needle track or beneath the skin (Figures 3, 4).

**Figure 5:**
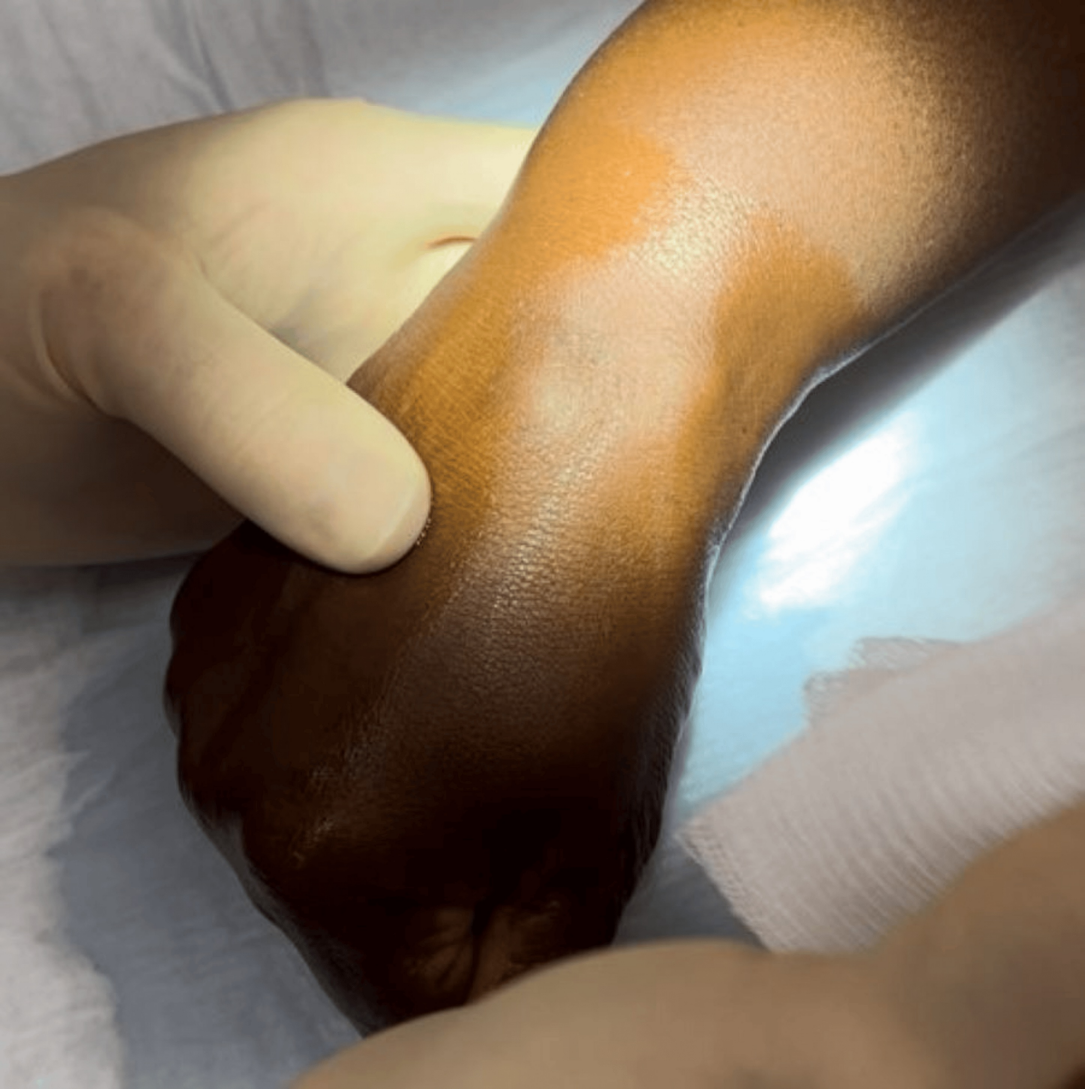
Decompressed dorsal carpal ganglion

Typically, one puncture is sufficient to fully decompress the ganglion however, if some fluid remains or the ganglion is multilobulated a second or third puncture and expression of the fluid may be attempted. Once the ganglion is fully decompressed (Figure 5), a band-aid is applied as well as an ace wrap that is meant to be worn for 24 hours. No immobilization is prescribed. The procedure generally takes less than 5 seconds to perform.

Data collection

A chart review was performed to collect basic demographic data (age, sex, hand dominance), location of ganglion, duration of symptoms, recurrence, any noted complications, prior treatment, and subsequent treatment. Patients were then contacted via telephone to confirm the above-collected data and asked several questions in relation to their ganglion. Patients were asked: "Did your ganglion come back at any time after the procedure?" "What was your satisfaction with the procedure on a scale of 1-10?" "A higher score would indicate more satisfaction. What is your current pain score on a scale of 0-10 with 0 being no pain and 10 being severe pain?" They were then asked several yes or no statements: “The procedure was painful,” “I would have the procedure for another ganglion,” and “I look favorably upon the procedure.”

Statistical analysis

Baseline comparisons between patients who did and did not have ganglion recurrence were performed using Student t-tests or Mann-Whitney U-tests for continuous variables and Chi-square tests for categorical variables. Multivariate analysis was deferred as only the location met the criteria for inclusion (p < 0.2 on the univariate analysis). Patient-reported outcome variables including a favorable view on aspiration, the likelihood of choosing aspiration again, pain with aspiration, satisfaction with aspiration, and pain at follow up were compared between patients who did and did not have ganglion recurrence using Mann-Whitney U-tests and Chi-square tests for continuous and categorical variables, respectively. Statistical significance was defined as p< 0.05. Statistical analyses were performed in R (version 4.3.2, R Foundation for Statistical Computing, Vienna, Austria).

## Results

Sixty-one patients were eligible and identified as having undergone the puncture procedure for a hand or wrist ganglia during the defined study period. All patients were attempted to be contacted via telephone and 34 patients were able to be reached to participate for an overall response rate of 56%. The mean age of patients who participated was 56.3. There were 12 (35.3%) male patients and 22 (65%) female patients. The mean duration of symptoms prior to the procedure was 28.3 months with a median of 3.75 months and a range of 0-360 months. The average length of follow-up from the date of the procedure to the time of the phone call was 38.3 months with a range of 28-52 months.

A total of 19 patients stated that their ganglion had returned for an overall recurrence rate of 55.9%. The mean time to recurrence after the procedure was 43 weeks with a median of 36 weeks. There were 11 dorsal carpal ganglia, four volar carpal ganglia, nine flexor sheath ganglia, and 10 mucous cysts included. Of the 34 patients, nine (26.5%) felt the procedure was painful, 27 (79.4%) would have the procedure for another ganglion and 26 (76.5%) said they look favorably upon the procedure. The mean overall satisfaction with the procedure was 8.35/10 and the mean current pain at the ganglion site was 0.44/10. No complications were reported.

Results comparing pre-procedure variables and follow-up outcomes between patients who did and did not have recurrence are shown in Table 1 and Table 2.

**Table 1:** Univariate analysis of 34 patients who underwent in office ganglion puncture by recurrence. Student’s t-tests and Mann-Whitney U-tests were used for continuous variables based on normality, and Chi-square tests were used for discrete variables. IQR interquartile range, SD standard deviation.

	Recurrence		Test statistic	P value
	Yes (19/34)	No (15/34)		
	55.88%	44.11%		
Age, years (mean ± SD)	55.9 ± 17.8	56.3 ± 15.3	t = 0.13	0.9
Sex				
-Male	7	5	χ^2^ < 0.001	1
-Female	12	10		
Follow-up, months (mean ± SD)	37.7 ± 7.3	39 ± 5.9	W = 163	0.49
Duration prior to intervention, months (median (IQR))	2.5 (1.75 – 24)	4 (2 – 12)	W = 127	0.92
Location				
-Flexor sheath	2	7	χ^2^ = 8.1	0.048
-Dorsal carpal	6	5		
-Volar carpal	4	0		
-Mucous	7	3		

**Table 2:** Patient reported outcomes of 34 patients who underwent in office ganglion puncture by recurrence. Mann-Whitney U-tests were used for continuous variables, and Chi-square tests were used for discrete variables. SD: standard deviation.

	Recurrence		Test statistic	P value
	Yes (19/34)	No (15/34)		
	55.88%	44.11%		
Complications	0	0		
Satisfaction with procedure (1-10), mean ± SD	7.25 ± 3.1	9.67 ± 1.0	W = 204	0.0049
The procedure was painful.	6/19	3/15	χ^2^ = 0.14	0.71
I would have the procedure for another ganglion.	12/19	15/15	χ^2^ = 4.9	0.027
I look favorably upon the procedure.	11/19	15/15	χ^2^ = 6.1	0.014
Current pain (0-10), mean ± SD	0.58 ± 1.1	0.27 ± 0.59	W = 129	0.54

Flexor sheath ganglia were statistically significantly less likely to recur with the procedure compared to all the other ganglia (p = 0.048). The patients who had no recurrence were more satisfied, more willing to undergo the procedure for another ganglion, and looked more favorably upon the procedure compared to those who did have recurrence (p < 0.01). There were no other significant differences between the two groups.

Of the 19 recurrences, eight (42%) had another intervention on their ganglion at the time of the phone call. Four patients had surgery and none recurred. The other four patients had another puncture procedure; three recurred and one did not recur.

Only six out of the 34 patients stated they had prior treatment on their ganglion before presenting to the senior author’s office for evaluation and subsequently undergoing the puncture procedure. Three of those patients’ ganglia recurred and three did not after the procedure.

## Discussion

Treatment of hand and wrist ganglia has changed little over the centuries. Observation, aspiration or puncture, and surgical excision remain the mainstays of treatment. It is important to reassure patients that the hand and wrist ganglia are not cancer and can spontaneously regress at times. Multiple studies have shown that some ganglia will spontaneously resolve with observation. The pediatric population has a high rate of resolution with observation with one study showing a 63% rate of spontaneous resolution [13] and another showing 79% [14]. In the adult population, Dias et al. showed in a prospective study a 42% rate of resolution of dorsal carpal ganglia with observation at a mean of 70 months [15]. Dias and Buch also prospectively showed a 53% spontaneous resolution of volar carpal ganglia at a five-year follow-up [16]. Despite around half of carpal ganglia resolving with observation, half persist several years later which can be a nuisance for patients.

Operative intervention remains the best option for minimizing recurrence rates; however, it is also associated with higher costs and associated morbidity [17]. Angelides and Wallace showed a <1% recurrence rate with open excision, but no other studies have been able to duplicate their success [7]. Other studies have shown a 39% recurrence rate for dorsal carpal ganglia [15] and a 42% recurrence rate for volar carpal ganglia [16]. Konigsberg et al. found a 6.8% recurrence rate for open excision and a 16.7% recurrence rate for arthroscopic with an average follow-up of 93 days [18]. McEvedy aggregated multiple studies from the early 1900s and reported a 24% recurrence rate with open excision [3]. A meta-analysis of two randomized control trials and four cohort studies showed a 21% recurrence rate for open excision and 6% for arthroscopic excision with higher morbidity for both than aspiration [17].

Various in-office procedures have been described for ganglia with a wide range of recurrence rates. Zubowicz and Ishii performed a prospective study over an eight-month period following 62 patients with mainly volar and dorsal carpal ganglia who underwent aspiration [11]. After one aspiration they reported a 74% success rate and increased this to 85% after three aspirations. Others have attempted adjuncts with steroids or sclerosants in addition to aspiration. Paul and Sochart had 89% “excellent or good” results with aspiration and injection of methylprednisolone and hyaluronidase versus 57% with aspiration and methylprednisolone only [19]. They concluded that the addition of hyaluronidase led to improved outcomes. No other studies have been able to reproduce the successes of these studies and others have raised concerns regarding the morbidity with sclerosants such that sclerosants are no longer recommended in treating ganglia [12].

Richman et al. performed a prospective study on 87 consecutive digital and carpal ganglia that underwent aspiration and multiple punctures with or without immobilization [9]. The overall success rate was 36%. Those who had three weeks of immobilization had a success rate of 43% versus 29% of those who mobilized immediately. They concluded that immobilization was helpful in reducing recurrence rates. Korman et al. duplicated the protocol of Richman et al. and found no difference between immobilization and early mobilization with overall recurrence rates of around 50% [1,9]. Other studies have compared aspiration with or without steroids. Varley et al. randomized wrist ganglia with either aspiration alone or aspiration with an injection of methylprednisolone [10]. Both groups roughly had a 33% success rate in treating ganglia and they concluded that the addition of steroids was of no benefit and ran the risk of skin depigmentation and fat atrophy. Yet another study compared aspiration versus aspiration with multiple punctures of the needle and found a success rate of 31% with aspiration alone versus 22% with aspiration and multiple punctures [20]. The study concluded that multiple punctures did not improve outcomes [20]. A meta-analysis using randomized control trials and cohort studies from 1990 to 2013 showed a 59% recurrence rate with aspiration [17].

These studies all highlight the confusion in the literature surrounding aspiration and puncture for the hand and wrist ganglia. There is clearly conflicting literature regarding immobilization and the use of steroids. However, when there is conflicting evidence it is likely best to proceed with the simpler treatment with less morbidity which in our opinion would be aspiration or puncture alone without steroid or immobilization. We find the puncture procedure to be faster and more effective in fully decompressing ganglia than aspiration. The high viscosity of the mucinous fluid makes aspiration difficult through a small gauge needle and uncomfortable for the patient with a larger gauge needle. By firmly compressing the cyst and then puncturing it, the fluid will either erupt through the needle track or disperse subcutaneously.

Our results demonstrate a 55.9% recurrence rate which is consistent with the meta-analysis by Head et al. [17]. We also had no complications in our series and we found a high satisfaction rate with our patients even amongst those that experienced recurrence. There was also a statistically significant lower recurrence rate amongst flexor tendon sheath ganglia compared to all other ganglia which seems to be a consistent result shown in the literature. Korman et al. and Richman et al. also found a higher treatment success with the aspiration of flexor sheath ganglia [1,9]. Dearden et al. and Wang and Hutchinson showed a higher rate of spontaneous resolution of flexor sheath ganglia compared to carpal ganglia [13,14]. We hypothesize the treatment success of flexor sheath ganglia compared to other ganglia may have something to do with the relatively smaller size compared to carpal ganglia that may allow them to involute more easily and anatomical differences and rates of synovial fluid production of the flexor sheath compared to joints.

It is unknown why some ganglia recur with aspiration or puncture and others do not. Dias et al. and Dias and Buch compared observation, aspiration, and surgical excision of dorsal carpal ganglia and volar carpal ganglia in two prospective studies [15,16]. They concluded that neither aspiration nor surgical excision offered a clear benefit to the natural history of dorsal carpal ganglia and that the only long-term benefit of excision or aspiration of volar carpal ganglia over observation is the patient’s perception of its unsightliness. McEvedy proposed three stages in the life cycle of a ganglion: a stage of formation, a stationary phase, and a stage of diminution [3]. He described the stage of diminution as the period when a ganglion may gradually disappear or fail to reappear if ruptured [3]. It is plausible to suggest that ganglia that would have otherwise gradually resolved at the end of their life cycle are those that are successfully treated with aspiration or puncture. This may be why Dias et al. and Dias and Buch found no clear difference in long-term recurrence of aspiration versus observation for carpal ganglia [15,16]. However, the study by Dias et al. did show that patients were more satisfied with treatment whether it be aspiration or surgical excision compared to reassurance [15].

The puncture procedure is also helpful in the diagnosis of ganglia. A feared complication of ganglion excision is an unplanned resection of a sarcoma. Crosby et al. described a series of four patients who underwent unplanned resections of soft tissue sarcomas that were mistaken for dorsal carpal ganglia [21]. They recommended attempting to transilluminate the mass first. If it transilluminated, the mass could be treated as a ganglion. If it did not transilluminate then they recommended aspiration. If aspiration revealed mucinous, jelly-like fluid characteristic of a ganglion then it had to be treated as such; however, if there was no return of fluid, then advanced imaging with an MRI would have to be pursued. Some caution should be taken when transillumination is used as a diagnostic for ganglia. Daines and Strauch discussed two solid tumors of the hand that transilluminated on examination [22]. They were excised with pathology revealing a schwannoma and a neurofibroma. They hypothesized solid tumors high in water content can also transilluminate [22]. Therefore, we think a combination of both transillumination and a confirmatory test with puncture rather than transillumination alone is helpful in making the correct diagnosis. If no fluid is returned upon puncture, then an MRI is warranted for further workup.

There are several limitations to this study. There was a large percentage of patients who were unable to be reached via telephone to participate in the study limiting sample size and potentially leading to selection bias. This study design also introduces recall bias and response bias.

Overall our series demonstrated a recurrence rate of 56% of hand and wrist ganglia with no complications and high satisfaction. Flexor sheath ganglia have a lower recurrence rate compared to the other ganglia with simple puncture which is a consistent finding across the literature.

## Conclusions

In conclusion the puncture procedure is helpful in confirming the diagnosis of a ganglion while successfully providing definitive treatment in nearly half of patients. The literature is ripe with mixed evidence regarding optimal treatment of hand and wrist ganglia and how to best counsel patients. Flexor sheath ganglia have a lower recurrence rate compared to the other ganglia with simple puncture which is a consistent finding across the literature. Caution should be taken when attempting puncture of volar carpal ganglions to avoid injury to the radial artery. Our study supports that an in-office simple needle puncture procedure has a low complication rate with high patient satisfaction and is a reasonable first step in treating patients with suspected ganglia.
